# The Expression of Myoz1 and ApoB is Positively Correlated with Meat Quality of Broiler Chicken

**DOI:** 10.1155/2022/3266076

**Published:** 2022-12-31

**Authors:** Fatchiyah Fatchiyah, Rista Nikmatu Rohmah, Lidwina Faraline Triprisila, Regina Putri Virgirinia, Bayu Rahayudi, Nia Kurnianingsih, Anna Safitri, Ahmad Faizall Abdul Razis

**Affiliations:** ^1^Research Center of Smart Molecule of Natural Genetics Resource, Brawijaya University, Malang, East Java, Indonesia; ^2^Department of Biology, Faculty of Mathematics and Natural Sciences, Brawijaya University, Malang, East Java, Indonesia; ^3^Biosains Institute, Brawijaya University, Malang, East Java, Indonesia; ^4^Faculty of Computer Science (FILKOM) University of Brawijaya, Malang, East Java, Indonesia; ^5^Department of Physiology, Faculty of Medicine, Brawijaya University, Malang, East Java, Indonesia; ^6^Department of Chemistry, Faculty of Mathematics and Natural Sciences, Brawijaya University, Malang, East Java, Indonesia; ^7^Natural Medicines and Products Research Laboratory, Institute of Bioscience, Universiti Putra Malaysia (UPM), Serdang 43400, Selangor, Malaysia; ^8^Laboratory of Food Safety and Food Integrity, Institute of Tropical Agriculture and Food Security, Universiti Putra Malaysia (UPM), Serdang 43400, Selangor, Malaysia; ^9^Department of Food Science, Faculty of Food Science and Technology, Universiti Putra Malaysia (UPM), Serdang 43400, Selangor, Malaysia

## Abstract

Broiler chicken (*Gallus gallus*) is a source of animal protein with a high nutritional content. The purpose of this study was to evaluate the quality of broiler chicken meat (*Gallus gallus*) by analyzing its nutritional value, genetic profile, and protein level. The chicken meat samples were obtained from four different districts in Malang city, Indonesia. We analysed the proximate composition of chicken meat to detect its nutrition content. Furthermore, we have examined the sequence of the Myoz1 gene as well as the level of ApoB proteins in the same meat. The nutritional analysis of chicken meat showed that in the four locations different levels of protein, ash, water, and lipids were observed. The Myoz1 gene of femur chicken broilers from the second and third districts has five and twenty-one substitution mutations, respectively. The ApoB expression level in locations 2 and 3 was higher than that in the other districts. In conclusion, Myoz1 and ApoB levels were correlated with the nutritional content and quality of broiler chicken meat.

## 1. Introduction

Indonesian people have increased awareness of the importance of animal protein for the growth of body tissues. Indonesia, especially the Malang area, is the area with the largest number in the broiler industry sector. The main advantage of chicken meat is that it has a high nutritional value or content so that it is able to meet the nutritional needs of the organism. In addition, broiler chickens display a rapid body weight gain, the meat is tender, and the efficiency of feed is relatively high which means that the most of the feed can be easily converted into meat and is easily available in traditional and modern markets.

There are many intrinsic and extrinsic factors that affect meat quality, including muscle properties, such as muscle diameter [1], muscle growth [2], and muscle fiber type [3]. Muscle types can contribute to different metabolic rates in chickens due to different properties regarding oxidative capabilities, which have a large impact on feed conversion ratios. At the same time, the type of muscle fiber is also associated with the stability of meat color, tenderness, and water retention capacity. In the previous decade, both layer and broiler industries have developed rapidly, and broiler farming's target has shifted from increasing quantity to increasing quality as meat quality has become one of the most important goals for satisfying consumer preferences [4]. One of the selection marker genes that determine meat quality is Myoz1 [5]. There are three members of the myozenin (Myoz) family, including myozenin 1 (Myoz1), myozenin 2 (Myoz2), and myozenin 3 (Myoz3) that encode the proteins calsarcin-2 (FATZ1), calsarcin-1 (FATZ2), and calsarcin- 3 (FATZ3). Myoz1 is also reported as a candidate gene for determining heart failure and hypertrophy. Myoz1 expression increased in rats that had frequent exercise due to changes in muscle fiber type. In addition, the Myoz1 gene expression was also assessed in two different chicken variants, and it was found that the Myoz1 gene was not expressed in fast-twitch fiber chickens and that its expression was associated with tissue, sex, and age [6].

The fat content of chicken meat also contributes to the determination of meat quality since excessive fat can affect meat flavour. According to Fisher and Ginsberg [7], lipid accumulation in chicken broiler liver exposed to chronic heat stress can be determined from the ApoB threshold value. Apolipoprotein B (ApoB) is one of the hydrophobic proteins that has the highest molecular weight. This is the main structural protein of the triglyceride-rich low-density lipoprotein (LDL) receptor which plays important roles in transport and metabolism of lipids and energy [8, 9]. It has been reported that the level of ApoB is associated with body weight, abdominal fat, growth-related traits, and the meat quality of poultry [10, 11]. The broiler chicken contains 4536 ApoB amino acid residues which affect the growth and development of fat and have an impact on energy absorption and reproduction occupation. The aim of this study is to determine meat proximate analysis, genes profile (Myoz1), and protein expression level (ApoB) in samples from different district in Malang city, as well as to analyze the relation determining the possible candidate as selection markers in meat chicken quality.

## 2. Materials and Methods

### 2.1. Meat Samples

The samples of femur broiler chicken were obtained from 4 subdistricts in the Malang city. The samples are grouped into BR1Fem for chicken meat obtained from district 1, BR2Fem for chicken meat obtained from district 2, BR3Fem for chicken meat obtained from district 3, and BR4Fem for chicken meat obtained from district 4. Three meat samples were isolated from each district.

### 2.2. Proximate Analysis

The proximate analysis of all chicken meat samples was analysed at Saraswanti Indo Genetech Ltd., Indonesia, by 18-8-31/MU/SMM-SIG (Kjeltec) methods for protein, SNI 01-2891-1992, 6.1 for ash, 18-8-5/MU/SMM-SIG point 3.2.2 (Weibull) total lipid, SNI 01-2891-1992, and point 5.1 for water content.

### 2.3. DNA Extraction and PCR

Genomic DNA was isolated from the femur muscle by the standard method according to [12] with some modifications. The DNA concentration and purity were determined by a NanoDrop spectrophotometer, and the quality of the DNA was analysed using agarose gel 1%. The PCR mix solution composition was referred to [12]. The Myoz1 gene primer at exon 3 were F: 5′-AGGACCAAACCCTGCAAATG-3′; R: 5′-CCCTAAGAGTAAGACTGGCACAAG-3′ [5] with the following procedure: hot start at 95°C for 10 min; 35 cycles at 95°C for 30 s; annealing at 60°C for 30 s, and extension at 72°C for 40 s; and then postextension at 72°C for 10 min. The quality of the PCR product was analysed using agarose gel 1.5% and visualized using ChemiDoc gel imaging (Bio-Rad).

### 2.4. DNA Sequencing and In Silico Analysis

The PCR product was purified using Wizard® SV Gel and the PCR Clean-Up System (Promega). The product was sequenced by an ABI-Prims 3730xl DNA Sequencer (Köln, Germany), and the sequencing results were aligned with BioEdit software.

### 2.5. Western Blotting Analysis

Expression of APOB and *β* actin was analysed at the protein level in chicken meat. Protein of femur meat samples was separated in polyacrylamide gradient gels and then blotted to polyvinylidene fluoride (PVDF) transfer membrane (UltraCruz® PVDF Transfer membrane). The nonspecific protein blocking was applied to 5% skim milk in phosphate buffered saline (PBS) for an-hour's incubation at room temperature. The blotted membranes were incubated with polyclonal rabbit anti-Apolipoprotein B Polyclonal Antibody (Bioss, USA) and mouse anti-*β* actin (SantaCruz-Biotech., USA). The membranes were incubated with polyclonal AP-conjugated goat anti-rabbit IgG and goat anti-mouse IgG secondary antibodies (ThermoFisher Scientific, USA). The membrane was exposed using BCIP/NBT Membrane Alkaline Phosphatase Substrate (Rockland, Limerick, PA, USA) and visualized by the ChemiDoc imaging system (Bio-Rad Laboratories Inc, USA). The intensity of a specific protein was measured by Quantity One software.

### 2.6. Statistical Analysis

Statistical analysis was conducted using the GraphPad Prism5 statistical package (Graphpad software, USA). The one-way analysis of variance (ANOVA) followed by Tukey's HSD test was used to determine the significant difference of the data. *p* < 0.05 was considered significant.

## 3. Result

### 3.1. Proximate Composition

In this study, we performed proximate analysis to examine the levels of protein, ash, water, and lipids in broiler chicken meat isolated from 4 different locations. The result showed that broiler chicken meat at location 1 had a higher level of water (75.93 ± 0.04%) and ash content (1.35 ± 0.27%), and also a lower level of lipid (0.02%) than meat from other locations (Table 1). Importantly, we found a high level of protein in broiler chicken meat from location 1 (23.1 ± 0.04%) and location 3 (23.4 ± 0.16%).

### 3.2. Identification of Sequence Mutation of the Chicken Myoz1 Gene

**Figure 1:**
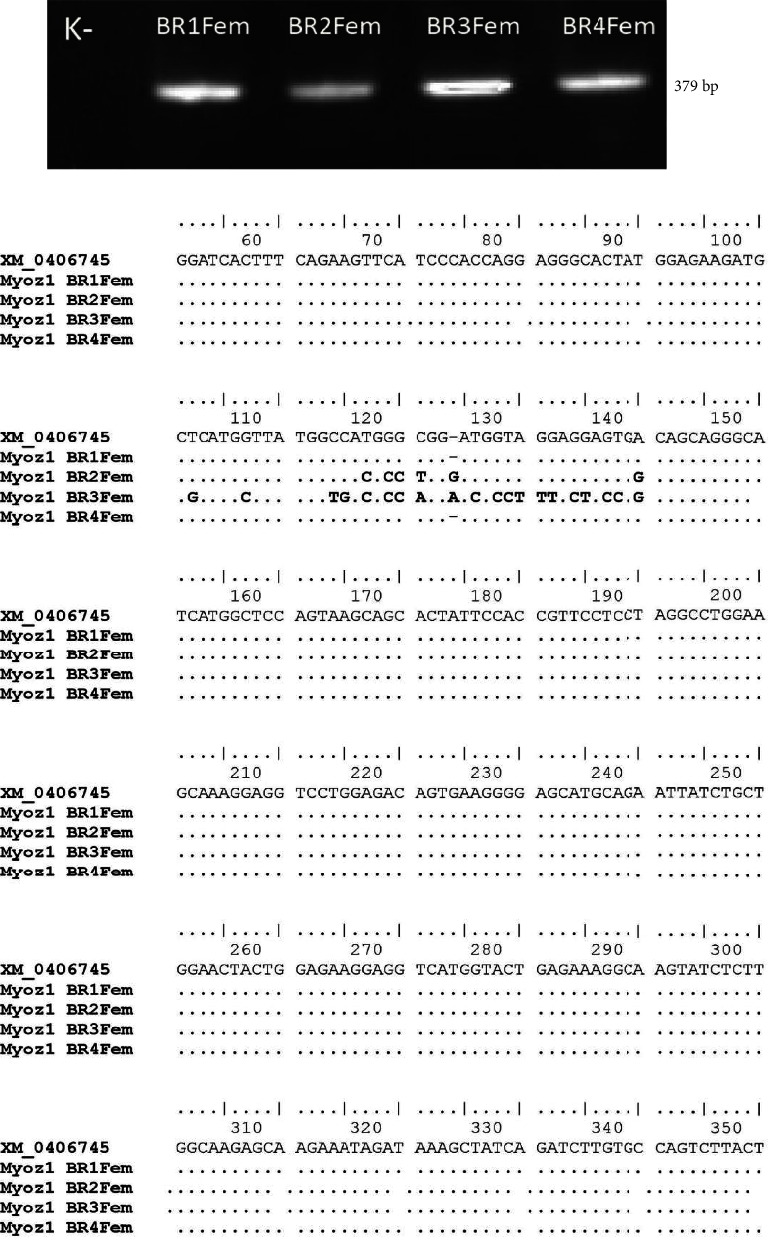
The profile of Myoz1 gene expression of broiler chicken femur from four different locations. (a) The amplicon of Myoz1 gene in 1.5% agarose gel. (b) Alignment of exon 3 of Myoz1 gene from the GenBank (XM_0406745) and broiler chicken from four locations (BR1Fem from district 1; BR2Fem from district 2; BR3Fem from district 3; and BR4Fem from district 4).

To identify the presence of mutations in the Myoz1 gene of broiler chicken meat, we amplify the DNA sequence of Myoz1 and then subject it to sequencing analysis. The amplification of the broiler Myoz1 gene was identified using PCR, and the DNA band profile was observed at 379 bp (Figure 1(a)). The results of the sequence alignment of four Myoz1 amplicons are shown in Figure 1(b). We identify the substitution mutations in Myoz1 gene of BR2FEM (T_117_-C_117_, G_119_-C_119_, G_120_-C_120_, C_121_-T_121_, T_140_-C_140_) and BR3FEM (T_102_-G_102_, G_107_-C_107_, C_114_-T_114_, C_115_-G_115_, T_117_-C_117_, G_119_-C_119_, G_120_-C_120_, C_121_-A_121_, T_125_-C_125_, C_121_-A_121_, T_126_-C_126_, G_128_-C_128_, T_129_-C_129_, A_130_-T_130_, G_131_-T_131_, G_132_-T_132_, G_134_-C_134_, G_135_-T_135_, G_137_-C_137_, T_138_-C_138_, and T_140_-C_140_). The insertion mutation was found in the Myoz1 gene of BR2FEM (G_23_) and BR3FEM (A_25_).

### 3.3. The ApoB Expression Level of Broiler Chicken

**Figure 2:**
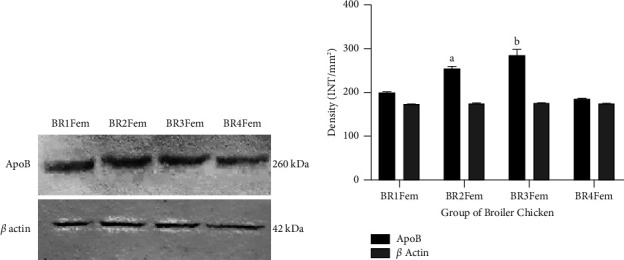
The level of ApoB protein of broiler chicken femur from four locations. (a) The expression profile of ApoB protein of broiler chicken isolated from 4 different locations. (b) The density of ApoB protein levels analysed by quantity one software. *β* actin was used as the loading control. *p* < 0.05, Tukey's HSD test.

In this study, the level of ApoB protein was measured using western blotting analysis of total proteins isolated from broiler chicken meat. The level of ApoB protein was significantly higher (*p* < 0.05) in BR2Fem (255.118 INT/mm^2^) and BR3Fem (284.596 INT/mm^2^) compared to BR1Fem (199.552 INT/mm^2^) and BR4Fem (186.213 INT/mm^2^) (Figure 2).

## 4. Discussion

The proximate analysis of broiler chicken meat obtained from 4 different locations showed some variations in the level of protein, ash, water, and lipid (Table 1). The differences between those parameters could be affected by the geographical location, diet, and hybrid used. These factors influenced the levels of protein, fat, ash, and water of chicken meat, thereby increasing variation in the quality of broiler chicken meat. Our results show that these four different locations provide meat of different quality, although they are still in the same city. Other factors that could affect the quality of chicken meat are rearing system, age, muscle pH, chemistry composition, muscle microstructure, postmortem aging, and processing methods [13]. The quality of chicken meat is also related to its composition, especially muscle protein, intramuscular collagen, and intramuscular fat content which are strongly influenced by breed, system maintenance, and the age of the chicken [13–16]. According to Dirong et al. [17], several methods can be applied to distinguish chickens from other species, including genomics, transcriptomics, proteomics, lipidomics, metabolomics, and glycomics, as well as the application of bioinformatics or chemometrics.

It has been reported that Myoz1 is expressed in fast-twitch skeletal muscles of humans, mice, and chicken [5, 6]. In this study, we analysed the sequence of the Myoz1 gene since its expression and single-nucleotide polymorphism are associated with the quality of chicken meat [5]. We found that chicken meat from the Myoz1 gene from locations 2 (BR2Fem) and 3 (BR3Fem) has substitution mutations. Previous studies have shown that mutations found in the chicken Myoz1 gene were associated with various traits including the weight of leg and breast muscle. Therefore, we hypothesized that mutations of this gene may affect the muscle weight and protein level of the chicken meat obtained from locations 2 (BR2Fem) and 3 (BR3Fem). It is known that Myoz1 deficiency decreases performance due to reduced body weight and fast-twitch muscles, resulting in excessive CaN/NFAT activation [6]. The NFAT activation can induce a muscle fiber type switch toward a slow-twitch and oxidative phenotype, related to the increased expression of a subset of genes associated with type I myofibers such as myoglobin and troponin I slow [18, 19]. We have previously reported that the substitution mutation of GAPDH gene sequences of chicken broilers can change amino acid sequences and the three-dimension structure of GAPDH protein [20]. Myoz family proteins are often used in the field of animal husbandry. Since the discovery of these three members, the Myoz family has emerged as one of the gene families most involved in the type of muscle fiber. In the pig species, all three members of the Myoz family were cloned and characterized [21], and the Myoz1 gene is a potential candidate as a marker to determine meat quality in animals. In addition, previous studies showed that Myoz1 was expressed in the liver, heart, breast muscle, and leg muscle of chicken, with a higher expression in both the leg and breast muscles than in the liver and heart [5]. The results of this study indicate that chicken meat from two locations has different quality and may have come from different breeds.

In our study, the ApoB expression level of BR2Fem and BR3Fem was high, suggesting that the lipid transport in the chicken muscle was accelerated and lipid accumulation was reduced [7]. This result indicates that both meat from BR2Fem and BR3Fem has a high quality. Several proteins that play an important role in determining intramuscular fat and meat quality include adipose differentiation related protein (ADFP), fatty acid transport protein 1 (FATP1), and apolipoprotein B (APOB) [11]. In conclusion, the meat qualities from 4 locations in the Malang area have a different composition which is influenced by environmental parameters and genotype. The Myoz1 gene of femur chicken broilers from the second districts and third districts has five and twenty-one substitution mutations, respectively. The ApoB expression level in locations 2 and 3 have a high level that correlated with lipid accumulation directly. Indeed, both Myoz1 and ApoB can be markers for determining the meat quality of chicken.

## Figures and Tables

**Table 1:** Proximate composition of femur broiler chicken from four locations.

	BR1Fem	BR2Fem	BR3Fem	BR4Fem
Protein (%)	23.1 ± 0.04	20.2 ± 3.1	23.4 ± 0.16	22.9 ± 0.42
Ash (%)	1.35 ± 0.27	0.95 ± 0.53	1.2 ± 0.014	1.09 ± 0.03
Water (%)	75.93 ± 0.04	73.95 ± 0.26	73.40 ± 0.15	71.03 ± 4.2
Lipids (%)	0.02 ± 0.01	1.05 ± 1.35	4.25 ± 0.28	2.62 ± 3.37

## Data Availability

All data generated during this study are available from the corresponding author on request.
